# Are there differences between stemless and conventional stemmed shoulder prostheses in the treatment of glenohumeral osteoarthritis?

**DOI:** 10.1186/s12891-015-0723-y

**Published:** 2015-10-01

**Authors:** Michael W. Maier, Sarah Lauer, Matthias C. Klotz, Matthias Bülhoff, David Spranz, Felix Zeifang

**Affiliations:** Clinic for Orthopedics and Trauma Surgery, Heidelberg University Hospital, Schlierbacher Landstraße 200a, D-69118 Heidelberg, Germany

**Keywords:** Stemless shoulder prosthesis, Osteoarthritis, Constant score, Proprioception, Joint position sense, Angle reproduction test

## Abstract

**Background:**

Conventional stemmed anatomical shoulder prostheses are widely used in the treatment of glenohumeral osteoarthritis. The stemless shoulder prosthesis, in contrast, is a new concept, and fewer outcome studies are available. Therefore, the purpose of the study was to investigate the early functional outcome and postoperative proprioception of a stemless prosthesis in comparison with a standard stemmed anatomic shoulder prosthesis.

**Methods:**

Twelve patients (mean age 68.3 years [SD ± 5.4]; 5 female, 7 male) with primary glenohumeral osteoarthritis of the shoulder were enrolled, who underwent total shoulder arthroplasty (TSA) with a stemless total shoulder prosthesis, Total Evolution Shoulder System (TESS®; Biomed, France). The control group consisted of twelve (age and gender matched) patients (mean age 67.8 years; [SD ± 7.1]; 9 female, 3 male), getting a TSA with a standard anatomic stemmed prosthesis, Aequalis® Shoulder (Tournier, Lyon, France). Patients were examined the day before and six months after surgery. The pre- and postoperative Constant Score (CS) was evaluated and proprioception was measured in a 3D video motion analysis study using an active angle-reproduction (AAR) test.

**Results:**

Comparing the postoperative CS, there was no significant difference between the groups treated with the TESS® prosthesis (48.0 ± 13.8 points) and the Aequalis® prosthesis (49.3 ± 8.6 points; *p* = 0.792). There was no significant difference in postoperative proprioception between the TESS® group (7.2° [SD ± 2.8]) and the Aequalis® group(8.7° [SD ± 2.7]; *p* = 0.196), either. Comparison of in the results of CS and AAR test pre- and postoperatively showed no significant differences between the groups.

**Discussion:**

In patients with glenohumeral osteoarthritis, treated with TSA, the functional and the proprioceptive outcome is comparable between a stemless and a standard stemmed anatomic shoulder prosthesis at early followup.

**Conclusion:**

Further follow-up is necessary regarding the long-term performance of this prosthesis.

**Trial registration:**

Current Controlled Trials DRKS 00007528. Registered 17 November 2014

## Background

In the surgical treatment of primary glenohumeral osteoarthritis, conventional stemmed shoulder prostheses are the golden standard and there are convincing results relating to pain loss and restoration of shoulder function after surgery [1–3]. The reason for developing new concepts such as stemless shoulder prostheses was that complications related to stemmed designs occurred, such as bone stock loss, intraoperative and postoperative periprosthetic fractures, mal-positioning of the humeral component, and in situations of infection more difficult eradication in the medullary canal [4, 5]. Therefore, stemless shoulder prostheses, such as the Total Evolution Shoulder System (TESS®; Biomed, France) were designed to reduce these potential risks associated with using a stemmed humeral implant. Today, stemless shoulder prostheses are increasingly being used, but only a few studies have reported the clinical results [1, 6–8]. After TSA, in order to use the replaced shoulder in activities of daily living, concerted interaction of the active stabilizers and the passive restraints of the replaced shoulder joint is necessary. It is known that not immobilization but active joint proprioception plays a considerable role in stabilization of the normal healthy shoulder and after different shoulder injuries by helping to control muscular action [9–11]. Current studies investigated shoulder proprioception in patients with glenohumeral osteoarthritis and the effect of a conventional stemmend total shoulder arthroplasty (TSA) on proprioception [12–14]. However, to date, no study has analyzed the postoperative proprioception of a stemless design. In stemless shoulder prostheses with potentially better reconstruction of the center of rotation, there might be a better proprioceptive outcome. Therefore, the study aim of the present study was, to compare the early functional outcome and postoperative proprioception of a stemless prosthesis with a standard stemmed anatomic shoulder prosthesis and to find out, if there are differences between the two prosthesis designs. The hypothesis was, that there are differences in functional and proprioceptive outcome between a stemless prosthesis and a standard stemmed anatomic shoulder prosthesis.

## Methods

Twelve consecutive patients (group STEMLESS) underwent TSA with the Total Evolution Shoulder System (TESS®) prosthesis for primary degenerative glenohumeral osteoarthritis with mean age of 68.3 years (standard deviation [SD] 5.4 years). The group comprised 5 women and 7 men (mean height 171.3 cm [SD 7.6]; mean weight 89.5 kg [SD 20.5]), with 5 right shoulders and 7 left shoulders. The dominant side was involved in 6 cases. The control group (CONTROL) consisted of twelve consecutive patients, underwent third-generation stemmed TSA (Aequalis Shoulder; Tornier, Lyon, France) for primary degenerative glenohumeral osteoarthritis with mean age of 67.8 years (standard deviation [SD] 7.1 years). This group included 9 women and 3 men (mean height 166.6 cm [SD 8.2]; mean weight 83.4 kg [SD 22.5]), with 9 right shoulders and 3 left shoulders. Informed consent was obtained from all patients. The dominant side was involved in 9 cases. Inclusion criterion was a primary glenohumeral osteoarthritis with an intact rotator cuff. Exclusion criteria were previous operations at the shoulder and rotator cuff tears.

### Operative techinque

All patients were operated by a single surgeon (FZ). In all shoulders, a deltopectoral approach was used as described by Neer et al. [15]. In no case was a rotator cuff tear found. After detachment of the subscapularis tendon and a capsular release, the joint was exposed. In all cases, the intraoperative joint status corresponded with the radiographic findings. The biceps tendon was dissected close to its glenoid attachment and was tenodesed in the bicipital groove in all cases. After placing of the implants, the subscapularis tendon was repaired by using three to five non-absorbable tendon-to-tendon sutures. To protect the reconstructed subscapularis tendon, the arm was placed in internal rotation in a shoulder abduction pillow for four weeks. Postoperatively, the shoulder was mobilized passively by a physiotherapist for six weeks to 60° of flexion and abduction and 0° of external rotation. Patients were asked to support these movements actively. Free range of motion was allowed six weeks after surgery.

All patients were evaluated preoperatively and six months postoperatively by using the Constant score (CS) [16, 17], adjusted for age and sex. Additionally, active range of motion was recorded for shoulder flexion, abduction, and rotation, with the hanging arm in a neutral position and the elbow flexed to 90°. Shoulder flexion, abduction, and external rotation were recorded in degrees, whereas internal rotation was graded according to the part of the spine that could be reached by the thumb. The CS was used to grade pain (with 0 points indicating severe pain and 15 points indicating no pain), activity (with 0 points indicating no mobility and 40 points indicating full mobility), and power (with 0 points indicating 0 kp [0 N] and 25 points indicating 12.5 kp [112.6 N]). Proprioception was measured one day before the operation and six months after surgery, using an active angle reproduction test as described previously [12, 13, 18]. All testing for this study was conducted at the Clinic for Orthopedics and Trauma Surgery, Heidelberg, by a single examiner. In accordance with the World Medical Association Declaration, the study protocol was approved by the local ethics committee (Ethics Committee Heidelberg), and informed consent was obtained from all patients and controls.

### Active angle reproduction test

**Fig. 1:**
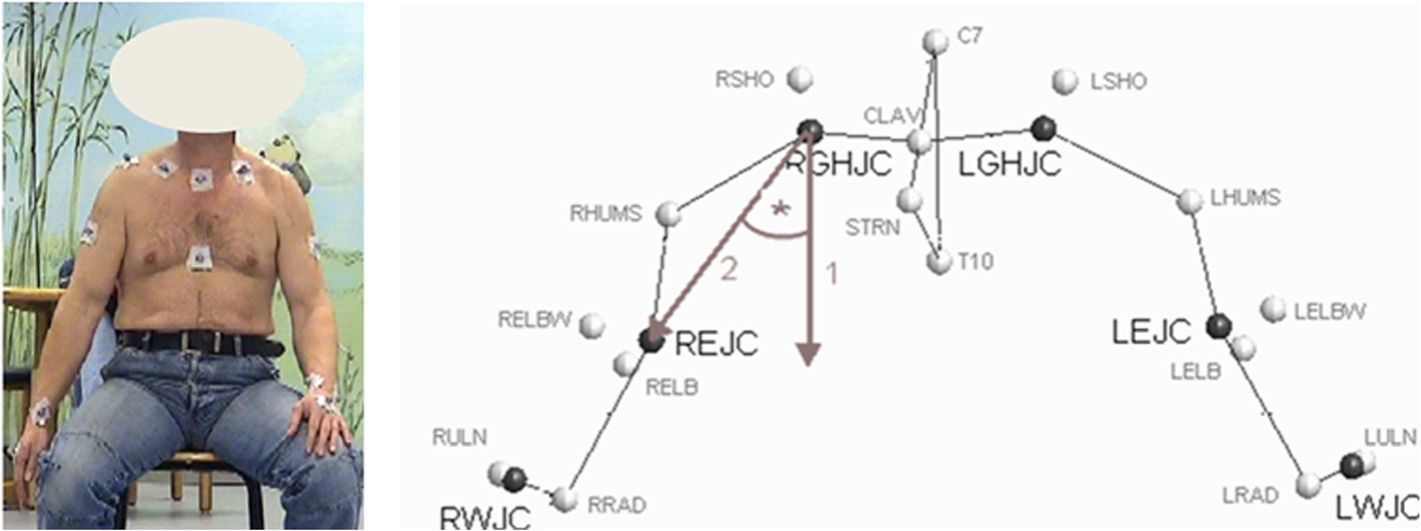
Left: Patient with markers for the three-dimensional motion video analysis (the patient gave specific written consent for the publiaction of their image). Right: Localization of the GHJC (glenohumeral joint center of rotation) and proprioception measurement in the ab-/adduction plane using the HUX model

A twelve-camera motion analysis system (Vicon 612; Vicon, Lake Forest, USA) operating at 120 Hz and the Heidelberg Upper Extremity (HUX)-model was used as described previously [19]. The spatial resolution of the system was approximately 1 mm. The HUX model consisted of seven segments: thorax, clavicles, upper arms, and forearms. The sternoclavicular and the glenohumeral joints were treated as a ball-and-socket joint, whereas the elbow was treated as a hinge joint. Translational degrees of freedom were not considered in any of these joints. For the measurement, the patients were prepared with four markers placed on the trunk as recommended by the International Society of Biomechanics [20]. Four markers were placed on each forearm: one at the radial and one at the ulnar styloid process of the wrist and two, connected with a wand, on the ulna close to the elbow joint (Fig. 1). For the AAR test, the patients sat on a chair with the arm hanging in 0° abduction and rotation. They were blindfolded to eliminate visual clues and wore sleeveless shirts. We ensured that the arm did not touch the trunk and, consequently, skin contact was minimized. The arm was moved to the desired position by the examiner with visual control of a manual, handheld goniometer. In detail, the positions were 30° and 60° abduction, 30° and 60° flexion, and 30° external (and afterwards 30° internal rotation) in 30° abduction (total of six joint positions). In the target position the subjects were told to maintain the position for ten seconds (in the meantime a mean value of the joint position was measured), and then the initial position with the arm hanging was resumed. Afterwards, the subject was asked to move the arm back into the target position. We measured the difference between the actual and the target joint position, and thus a smaller number indicates better proprioception. Standardized instructions were given to all subjects, and a test trial was conducted to acquaint them with each test condition. All tests were randomized for side and movement. Two test trials were performed at each angle, and the mean value was used for further analysis. The total proprioception performance (total) was defined as the mean value of all single measurements (six joint positions) to have one quality for comparing proprioceptive ability.

### Statistics

The statistical analysis was performed using SPSS Version 18.0 (SPSS Inc., Chicago, IL, USA). Group mean values (MV) and standard deviations (SD) were calculated. *P* values <0.05 were considered significant. The distribution of the data was checked with the Shapiro-Wilk test, and the homogeneity of variance was assessed using the Levene test. The angle between the long axis of the humerus and the trunk position was determined. Differences in shoulder joint angles between the target and reproduced position were compared between the pre- and postoperative examinations with a Wilcoxon-test for the groups. The Wilcoxon-test was also used to compare the pre- and postoperative CS and subscores. Differences among the groups were assessed by employing a Mann–Whitney *U* test.

## Results

In the present study, no intraoperative complications developed and no revision surgery was required after a mean duration of 6 months. The mean CS improved significantly in the STEMLESS group from 33.7 points (range, 9 to 61 points) preoperatively to 48.0 points (range, 25 to 65 points) postoperatively (*p* < 0.001), in the CONTROL group from 22.8 points (range, 15 to 31 points) preoperatively to 49.3 points (range, 35 to 62 points) postoperatively (*p* < 0.001). The preoperative and postoperative clinical examination findings are shown in Table 1. Preoperatively CS was significant worse for the CONTROL group (22.8 points (range, 15 to 31 points) vs. 33.7 points (range, 9 to 61 points) for the STEMLESS group; *p* = 0.038). There was no significant difference in CS postoperatively between the groups treated with the TESS® prosthesis (48.0 ± 13.8 points) and the Aequalis® prosthesis (49.3 ± 8.6 points; *p* = 0.792; Table 1).

**Table 1:** Comparison of the Constant score between the STEMLESS group and controls

	Group STEMLESS (TESS®)	Group CONTROL (Aequalis®)	*P* values
	*n* = 12	*n* = 12	
Preoperative			
Constant score (points)	33.7 ± 16.1 (9 to 61)	22.8 ± 5.8 (15 to 31)	0.038
Pain (points)	6.3 ± 3.8 (0 to 10)	5.0 ± 2.1 (0 to 10)	0.328
Power (points)	2.8 ± 4.2 (0 to 9)	0.7 ± 2.3 (0 to 8)	0.131
Activity (points)	7.6 ± 2.5 (5 to 13)	5.9 ± 1.4 (4 to 8)	0.061
Mobility (points)	17.0 ± 8.1 (4 to 30)	11.2 ± 4.8 (6 to 18)	0.042
Flexion (deg)	94.2 ± 27.5 (60 to 160)	80.4 ± 26.0 (40 to 120)	0.221
Abduction (deg)	79.6 ± 34.0 (25 to 170)	57.9 ± 18.6 (30 to 90)	0.066
External rotation (points)	4.3 ± 3.5 (0 to 8)	1.8 ± 2.9 (0 to 8)	0.069
Internal rotation (points)	3.7 ± 2.2 (0 to 6)	2.8 ± 2.2 (0 to 6)	0.363
Postoperative			
Constant score (points)	48.0 ± 13.8 (25 to 65)	49.3 ± 8.6 (35 to 62)	0.792
Pain (points)	11.7 ± 3.9 (5 to 15)	12.9 ± 3.3 (5 to 15)	0.408
Power (points)	4.4 ± 4.6 (0 to 10)	3.8 ± 4.8 (0 to 11)	0.766
Activity (points)	11.6 ± 3.2 (6 to 17)	11.3 ± 2.4 (8 to 15)	0.832
Mobility (points)	20.3 ± 5.9 (10 to 30)	21.2 ± 2.6 (16 to 26)	0.659
Flexion (deg)	96.9 ± 16.4 (73 to 127)	92.4 ± 16.5 (65 to 130)	0.509
Abduction (deg)	85.9 ± 20.7 (50 to 128)	86.2 ± 12.7 (72 to 116)	0.972
External rotation (points)	5.3 ± 3.4 (0 to 8)	6.2 ± 2.5 (2 to 8)	0.504
Internal rotation (points)	4.8 ± 1.0 (4 to 6)	5.3 ± 1.3 (2 to 6)	0.308

**Fig. 2:**
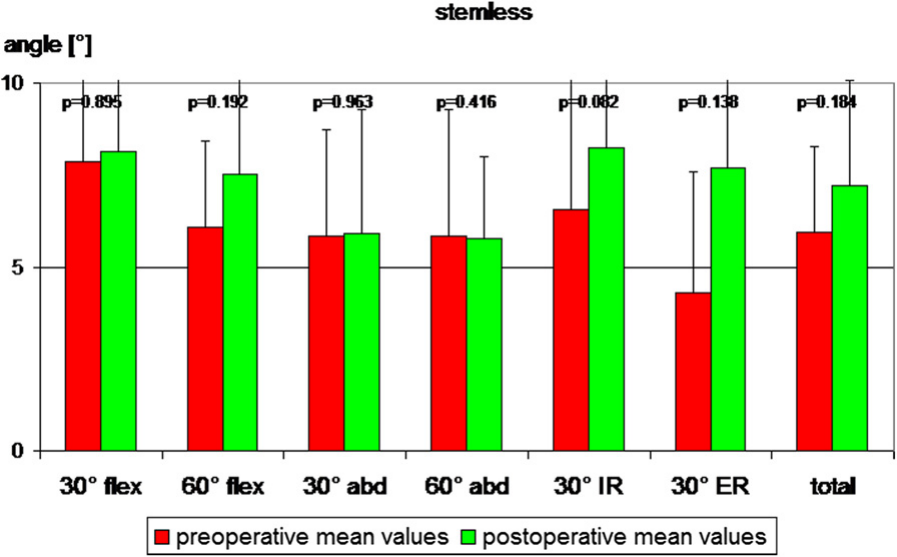
The stemless (TESS®) group displayed no significant differences between pre- and postoperative AAR

**Fig. 3:**
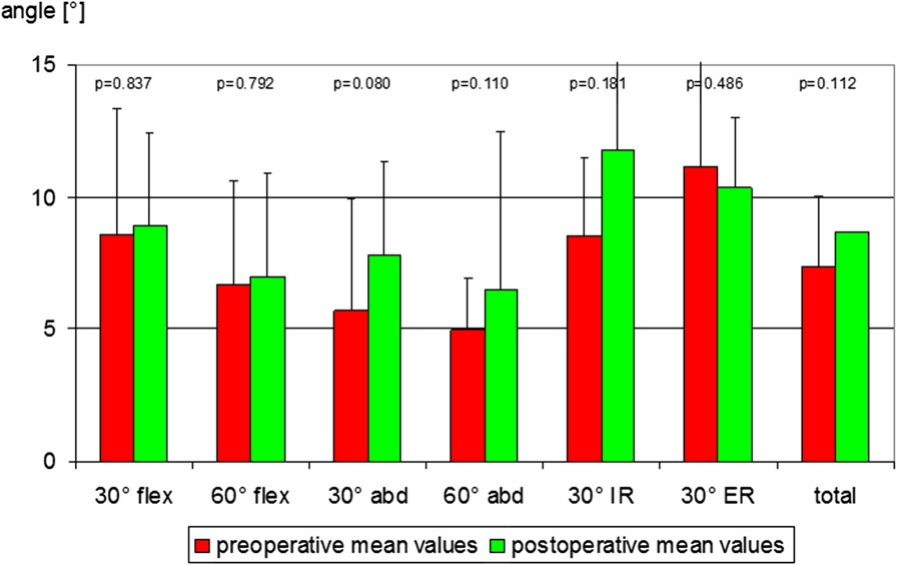
The control (Aequalis®) group displayed no significant differences between pre- and postoperative AAR

**Fig. 4:**
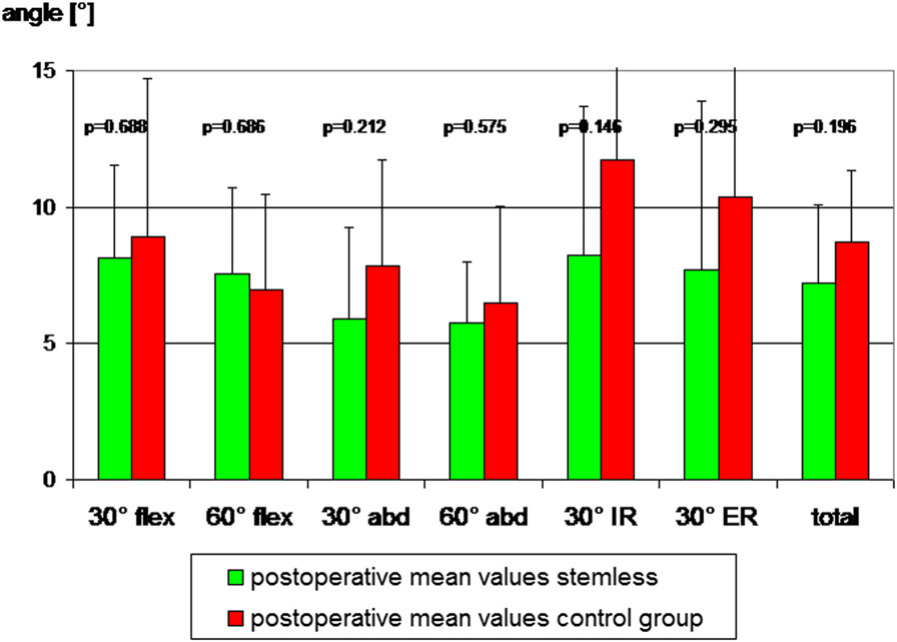
Comparison of postoperative proprioception between the stemless (TESS®) and the control (Aequalis®) groups showed no significant differences between the two groups

In the STEMLESS group the total proprioception, defined as the mean value of all single measurements (six joint positions), did not display any significant changes six months after surgery (Fig. 2). By trend proprioception had deteriorated in five of six single measurements. Only at 60° of abduction (60° abd) was no deterioration observed (Fig. 2). The CONTROL group also revealed no significant changes six months after surgery. By trend, proprioception had deteriorated in five of six single measurements (Fig. 2). Comparison of postoperative proprioception showed no significant difference between TESS® group (7.2° [SD ± 2.8]) and Aequalis® group (8.7° [SD ± 2.7]; *p* = 0.196; Fig. 3)). By trend, postoperative proprioception was better in the STEMLESS group in five of six single measurements (Fig. 3). Comparing the overall differences between pre- and postoperative AAR, there is no significant difference between the STEMLESS (1.3° [SD 3.1]) and CONTROL groups (1.4° [SD 2.7]; *p* = 0.935; Fig. 4; Table 2).

**Table 2:** Comparison of pre- to postoperative proprioception differences between the STEMLESS and the CONTROL group

	STEMLESS (TESS®)		CONTROL (Aequalis®)		*p*-value
Movement	Diff. [°]	SD [°]	Diff. [°]	SD [°]	
30° of flexion	0.3	±6.7	0.4	±6.1	0.967
60° of flexion	1.5	±3.4	0.3	±3.9	0.453
30° of abduction	0.1	±4.0	2.2	±3.8	0.194
60° of abduction	−0.6	±2.3	1.8	±2.8	0.061
30° of external rotation	3.4	±7.4	−2.2	±9.1	0.134
30° of internal rotation	1.7	±3.0	3.2	±6.9	0.510
Total proprioception	1.3	±3.1	1.4	±2.7	0.935

*Diff* pre- to postoperative differences, *SD* standard deviation; Positive values indicate a deterioration of proprioception

## Discussion

Today stemless shoulder prosthesis are increasingly being used, but only a few studies have reported about the clinical results [1, 6–8]. Therefore, the aim of the present study was to find out, if there are differences between the golden standard - a conventional stemmed shoulder prosthesis - and a stemmless prosthesis design in patients with primary glenohumeral osteoarthritis. The hypothesis was, that there are difference in functional and proprioceptive outcome, but the results of the present study show that in patients with glenohumeral osteoarthritis treated with TSA, no significant differences were found concerning the functional and proprioceptive outcome between a stemless prosthesis (TESS®) and a stemmed anatomic shoulder prosthesis (Aequalis®) at early follow-up.

In the literature, only few studies concerning stemless shoulder prostheses are available [1, 6–8]. Table 3 shows the literature results in comparison to the results of the present study. In our STEMLESS (TESS®) group, the CS improved significantly from 34 points preoperatively to 48 points six months after surgery. The relatively low postoperative CS is caused by the short follow-up period of six months compared to the other studies. The short follow-up is also responsible for the relatively small postoperative active flexion of 97° in our stemless cohort. Kadum et al. [6] also reported about the TESS® prosthesis in 56 consecutive patients who were operated with one of the two versions of TESS (anatomical or reverse). Here, 19 patients with primary/ posttraumatic osteoarthritis without cuff arthropathy were treated with an anatomical TESS prosthesis, comparable to our patients. They also reported on functional improvement in a short term follow-up of 14 months, with an significant improvement in the quick DASH and EQ-5D. In another study by Huguet et al. [7], 72 shoulders were treated with the TESS® prosthesis and 63 patients were reviewed. Their results showed a gain in active mobility of about 49° for forward flexion and 20° for external rotation after three years. The mean preoperative CS was 30 the postoperative CS was 75. They also concluded, that their clinical results were similar to those for classical prostheses. Humeral head removal facilitates glenoid exposure and implantation. Huguet did not see any specific complication in his short term follow-up, which is comparable to our results. Razmjou et al. [8] reported in a prospective two year follow-up study the clinical and radiologic outcomes of TSA using 3 different prosthetic designs, inter alia with the Neer II system a stemmed prosthesis, and the stemless TESS® prosthesis for patients with advanced osteoarthritis of the glenohumeral joint. They also found similar results for stemless and stemmed prosthesis with significant improvement in functional scores, ROM, and strength. Solely the active external rotation at 90° abduction was statistically significantly lower in the Neer II group. The incidence of lucent lines around the glenoid component was higher in the Neer II group. No statistically significant relationship was seen between type of prosthesis and patient satisfaction. In the present study, we did not find a significant difference in external rotation between stemless and stemmed prostheses (*p* = 0.504). The study of Berth et al. [1] confirms this results. They measured the CS, the DASH score, and the active range of motion for abduction, anteversion, and external rotation in 82 patients with primary osteoarthritis of the shoulder treated with either the TESS® prosthesis or the Affinis® stemmed shoulder prosthesis. Their patients were examined before and 32 months after surgery. No significant differences were found in CS. They showed an advantage of the stemless design over the stemmed design with a significantly lower estimated blood loss and lower mean operative time in the group with the stemless shoulder prosthesis.

**Table 3:** Studies reporting about stemless prostheses in patients with glenohumeral osteoarthritis without cuff arthropathy

Study	Implant	No	FU	Constant-Score [points]	Active flexion [degree]
Kadum et al.	TESS®	19	14	-	-
Huguet et al.	TESS®	63	36	30 → 75	96 → 145
Razmjou et a.	TESS®	17	24	-	69 → 135
Berth et al.	TESS®	41	32	30 → 55	81 → 116
Current Study	TESS®	12	6	34 → 48	94 → 97

No, number; FU, follow-up (months); TESS® = Total Evolution Shoulder System

The present study measured proprioception using an AAR test as described before [12, 13] and found no significant difference in proprioception between the STEMLESS and the CONTROL group. The results were comparable to published results of healthy 65 year old controls without shoulder pathology with mean proprioceptive performance of 7.8° in the AAR test [13]. By trend in the present study, with a mean failure of 7.2° in the AAR test, the STEMLESS group showed better postoperative proprioception than the stemmed CONTROL group with 8.7° but there was no differences comparing the pre- and postoperative differences between the groups (deterioration of 1.3° in the stemless and 1.4° in the control group; *p* = 0.935). Therefore, this might be related to the slightly different initial proprioceptive value. Therefore factors like surgical approach, operative time, soft tissue damage, rotator cuff status, bone injury, and reconstruction of the anatomical structure, potentially influencing postoperative proprioception have to be discussed.

Rokito et al. [21] investigated the degree to which the surgical approach affects the recovery of strength and proprioception and showed, that surgical approach and intraoperative soft tissue management could play an important role for the proprioceptive outcome. On the one side there might be a lower mean operative time in stemless shoulder replacement, which could be associated with less soft tissue damage and therefore better postoperative proprioception. On the other side, in the present study, in both groups the same deltopectoral approach was used as described by Neer et al. [15], patients with existing rotator cuff tears were excluded, and all patients were operated by a single surgeon. Due to the fact that proprioceptors are found in the periosteum and injury of the bone is more extensive with implantation of a stemmed prosthesis, this might be an indication for worse proprioception after stemmed prosthesis. Ingemarsson et al. [22] showed that after hip fractures, balance, stance control and active joint angle position are frequently damaged but till today there is no evidence about an intraoperative bone injury and proprioceptive outcome.

The philosophy of the TESS group [7], developing this prosthesis in 2003 was a stemless restoration of the anatomy of the proximal humerus with superior reconstruction of the humeral head geometry, especially of the humeral head fulcrum. While the stemless TESS prosthesis can be adapted to the humeral head geometry without any external restraints, the stemmed Aequalis® prosthesis allows only for limited adjustments as the inclination can only by modified in steps and the offset of the center of rotation can only be set along a simple eccentric track [23, 24]. Irlenbusch et al. [24] determined in vivo the individual humeral-head rotation centers from the position of the adjustable prosthesis taper and the eccentric head and showed that the range of pathologicoanatomical deviation is substantial. They concluded, that there is the need for an adjustable prosthetic system. Hypothetical, with a stemless TESS prosthesis, providing a better reconstruction of these anatomical structures, there is a better restoration of the center of rotation what might cause a better proprioceptive feedback of the rotator cuff muscles. But in conclusion, the present study did not show a significant proprioceptive difference between the stemless and stemmed design.

The present study has some limitations. The follow-up period (6 months) is relatively short. There was no randomization and matching of the patients according to age, height, weight, body-mass-index, gender or according to the dominance of their arm. We didn’t do a priori power analysis and reported only short-term follow-up. Nevertheless, the first few months after surgery are particularly important in terms of early postoperative rehabilitation and complication types and, there, the stemless TESS® prosthesis showed promising short-term results comparable to a conventional stemmed prosthesis. Long-term follow-up is required to confirm the results of this innovative system in the long term.

## Conclusion

In patients with glenohumeral osteoarthritis treated with a total shoulder arthroplasty, functional and proprioceptive outcomes are comparable between a stemless and a standard anatomic shoulder prosthesis at early follow-up. Further follow-up is necessary to assess the mid- and long-term performance of this prosthesis.
